# Off-pump versus on-pump coronary artery bypass grafting in acute coronary syndrome: a clinical analysis

**DOI:** 10.1186/1749-8090-5-31

**Published:** 2010-04-27

**Authors:** Kaan Kaya, Raif Cavolli, Alpaslan Telli, Mehmet Fazil Tolga Soyal, Alp Aslan, Gökhan Gokaslan, Şahin Mursel, Refik Tasoz

**Affiliations:** 1 Division of Cardiovascular Surgery, Ozel Ulus Hastanesi, Ankara, Turkey; 2 Division of Cardiovascular Surgery, Kavaklidere Umut Hastanesi, Ankara, Turkey; 3 Division of Cardiology, Ozel Ulus Hastanesi, Ankara, Turkey

## Abstract

**Background:**

Although off-pump coronary artery bypass (OPCAB) surgery has many beneficial effects compared with on-pump surgery, switch to on-pump surgery has significantly higher risks of operative mortality. Benefits of OPCAB over on-pump surgery strategies concerning myocardial revascularization are still debatable. We have aimed to develop an "algorithm of off-pump surgical strategy" on preventing conversion to on-pump. This clinical study reports our clinical outcome of OPCAB in patients with acute coronary syndrome.

**Methods:**

Between January 2006 and December 2008, 198 patients with acute coronary syndrome were enrolled in the study. Decision of OPCAB (142 patients) or on-pump surgery (56 patients) was made according to patients' present clinical status and our surgical background. Cardiac enzymes, duration of the surgery, graft numbers, stay in intensive care unit were recorded.

**Results:**

OPCAP group has shorter operation time (82.78 min versus 164.22 min, p < 0.001), lesser necessity for intra-aortic balloon pumping (3.5% versus 12.5%, p = 0.053), shorter duration of intensive care unit stay (p < 0.05) and hospital stay (p < 0.001) compared to on-pump patients. EuroSCORE level was lower in OPCAP group (p < 0.001). None of the patients of OPCAB group required conversion to on-pump technique.

**Conclusions:**

The patients who admitted to the hospital with acute coronary syndrome within "golden hours" (within 6 hours after onset) had a greater chance for OPCAB surgery. This study proves that EuroSCORE is likely to be an important factor in deciding which surgical technique to use, but a further investigation is needed to verify. According to our findings, a careful evaluation of coronary angiography, hemodynamic status, quality of target coronary vessel and timing of surgery are important for OPCAB surgery to avoid conversion to on-pump. By a careful systematic evaluation of the patients as explained with this article, it can be prevent or reduce conversion to on-pump surgery during OPCAB surgery.

## Background

Cardiopulmonary bypass (CPB) and cardioplegic arrest provide bloodless and immobile surgical field during coronary artery by-pass grafting surgery (CABG). However, developments in surgical instruments (stabilization devices, intra-coronary shunts, etc.) parallel to surgeons' experience has made off-pump coronary artery bypass surgery (OPCAB) widely accepted technique by an increasing number of cardiac centers. Nevertheless, OPCAB coronary surgery is not a "new" technique; Kolessov [1] and Favaloro [2] were reported their first results at the end of 1960's. Later, this technique was abandoned as the use of cardiopulmonary bypass and cardioplegic arrest became routine. CPB seemed to provide more comfortable surgical technique during cardioplegic arrest, but soon many problems were observed as its deleterious effects including "systemic inflammatory response syndrome" (SIRS), "post-pump syndrome", "post-perfusion syndrome" and "adult respiratory distress syndrome" (ARDS). These all problems mentioned above have been associated with multi-organ dysfunction involving cardiac, vascular, pulmonary, neurologic, renal, gastrointestinal and hematologic systems.

Because of the adverse effects of CPB mentioned above, OPCAB technique have regained interest by the lots of cardiac surgeons and increasing numbers of reports have been published during last decade all over the world. Ngaage has carefully reviewed advantages and disadvantages of off-pump coronary surgery [3], but he could not make a certain proposition if the OPCAB technique is better. Although OPCAB surgery is associated with lower mortality and morbidity compared with on-pump CABG [4-6], patients who required intra-operative conversion from off-pump to on-pump surgery have been reported to have a poorer outcome than patients having a successfully completed OPCAB surgery [7-10]. In a randomized clinical trial, Van Dijk and colleagues concluded that OPCAB is safe and yields a short-term cardiac outcome comparable to on-pump CABG in selected patients [11]. Although the patients operated via off-pump technique have better short-term outcomes, the aborted off-pump CABG patients have significantly higher risks of operative mortality and morbidity than those who completed off-pump [7,10]. Of course, none of the surgeons wants to convert from off-pump to on-pump surgery, since there is not a demarcated margin while deciding the surgical technique. Although there is some reports concerning the benefits, indications and results of OPCAB surgery in patients with evolving acute coronary syndrome requiring emergent surgery [12,13], there is not a standardized algorithm for feasibility analysis of performing OPCAB surgery.

**Figure 1:**
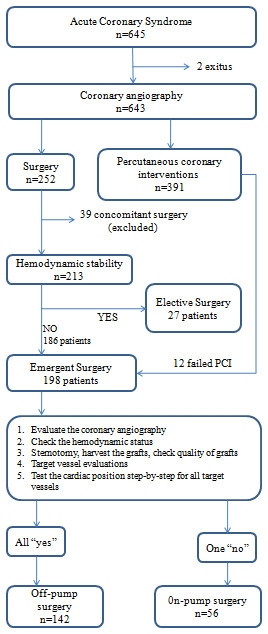
**Patient Allocation Diagram**.

**Figure 2:**
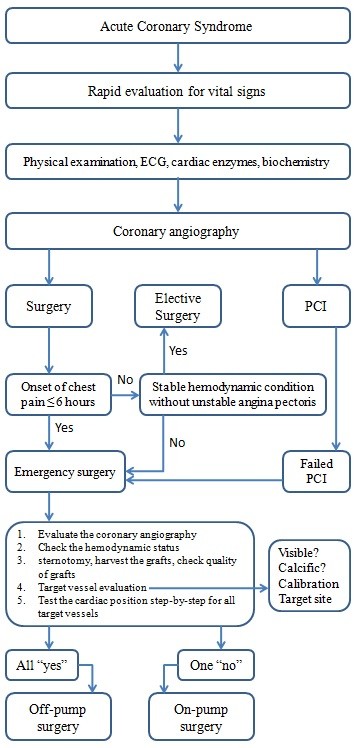
**The diagram of off-pump coronary artery bypass surgery selection criteria**.

The aim of the study was to report and compare our clinical results from emergent OPCAB and on-pump operations in patients suffering acute coronary syndrome. Eventually, we have aimed to form an "off-pump surgical strategy algorithm" to avoid conversion to on-pump technique in that risky patient group. Because, there is not any detailed investigation in literature about algorithm of OPCAB surgery previously, we operated our patients based on our experience in the past (Figure 1). At the end of the study, we succeeded to form our final off-pump surgical strategy algorithm (Figure 2).

## Methods

Since 2003, the same surgery team had operated over 2000 patients. Between January 2006 and December 2008, 645 patients were admitted to our emergency service with the diagnosis of acute coronary syndrome. Seven patients had cardiac arrest due to ventricular fibrillation immediately before coronary angiography, 5 survived and 2 of them died after cardiopulmonary resuscitation (Figure 1). 391 of 643 patients were destined to percutaneous coronary intervention (PCI) and medical treatment and the remaining were directed to coronary surgery. Together with 12 failed PCI patients, remaining 252 patients underwent to open heart surgery. Thirty-nine patients had concomitant procedures (mitral valve repair/replacement, aortic surgery etc.), 27 patients were operated electively and they were they were excluded from the study. 142 of 198 patients underwent off-pump and 56 had on-pump surgery (Figure 1). The preoperative data are shown in Table 1. We received informed consent from the patients and their family for cardiac surgery.

**Table 1:** Demographic variables of the patients.

	Off-pump (n = 142)	On-pump (n = 56)	Odds ratio	P value
**Age**	61.75 (± 11.4)	63.42 (± 9.1)	-	0.327
**Female**	24.6%	30.4%	1.333	0.474
**Body mass index**	25.34 (± 2.1)	25.11 (± 2.1)	-	0.486
**Diabetes**	35.2%	39.3%	0.840	0.625
**Smoke**	45.8%	39.3%	1.305	0.431
**Hypertension**	52.8%	48.2%	1.202	0.636
**Chronic obstructive lung disease**	4.9%	3.6%	1.400	1.000
**Peripheral arterial disease**	3.5%	3.6%	0.895	1.000
**Old myocardial infarction**	18.3%	10.7%	1.868	0.283
**Re-operation**	2.8%	0	0.972	0.579

### Anesthesia and surgical management

After monitorization of ECG, arterial blood pressure and peripheral oxygen saturation, every patient was administered a standard general anesthesia. 1.5 g of Cefuroxime was used for antibiotic prophylaxis. Anesthesia was induced with 0.05 mg/kg midazolam, 3 μg/kg sufentanil and 0.1 mg/kg vecuronium intravenously and maintained with a continuous infusion of 0.5 μg/kg/h sufentanil and 0.1 mg/kg/h propofol. A warm water blanket was used to manage the body temperature. Median sternotomy was performed. Intravenous heparin (100-150 IU/kg for OPCAB and 300 IU/kg for on-pump CABG) was administered just before completion of left internal thoracic artery (LITA) and saphenous vein graft (SVG) harvest. Pericardial sac was cut to hang with silk sutures. This is the point for evaluation of the patient to decide which technique should be chosen; off-pump or on-pump coronary bypass surgery.

Our decision parameters for off-pump surgery were;

1. A stable hemodynamic status: Some of patients can show a good hemodynamic status despite unstable angina pectoris and we can perform off-pump surgery without any problem. If their hemodynamic status is worsening despite medical supportive treatment, the off-pump coronary surgery most probably cannot be performed. If it is not possible to achieve an adequate hemodynamic condition despite adequate medication, then we performed on-pump surgery.

2. Target vessel evaluation: performed twice; preoperatively on coronary angiography and intra-operatively by naked eye and finger palpation.

a. Visual evaluation: A careful visual evaluation should be done for all of target vessels to see if they are all visible. Some patients, particularly obese patients may have a thick epicardial fat layer or some target vessels may take a deep course in the myocardium. Therefore, coronary arteries cannot be visualized easily, and performing an off-pump technique can be very difficult.

b. Calibration: Target coronary vessels should be evaluated for their caliber. A small-caliber target coronary vessel makes it difficult to perform a good off-pump anastomosing technique; especially presence of a small-caliber LAD is an important problem to perform an off-pump surgery as well as circumflex and right coronary arteries.

c. Quality: Target coronary vessels were evaluated for their quality: In some conditions, a coronary endarterectomy may be needed. In addition, off-pump coronary endarterectomy may yield good result. However, if predicted endarterectomy sites involve more than one vessel and/or is hard to approach to the vessel it may compromise quality of anastomosis more than on-pump surgery.

3. Graft evaluation: Calibration and quality of the grafts are also very important factors effecting anastomosing quality during coronary surgery. If patient has a serious graft problem, it will be better to perform on-pump surgery.

4. Heart positioning: Test hemodynamic status by positioning the heart. Especially manipulations to expose circumflex artery and its branches are more likely to have a depressive effect on hemodynamic status. Cessation of antihypertensive drugs before manipulation of the heart is of importance since positioning the heart for exposure of the circumflex arterial system is likely to reduce blood pressure below acceptable level. If hemodynamic parameters were deprived while doing exposure maneuvers, we performed on-pump surgery. In some cases, as some surgeons prefer, LITA to LAD anastomosis prior the other anastomoses may maintain cardiac performance, this allow to complete the revascularization without extracorporeal circulation.

Following stepwise evaluation described above we made a decision for each patient whether we should use on-pump or off-pump operation technique (Figure 2).

**Figure 3:**
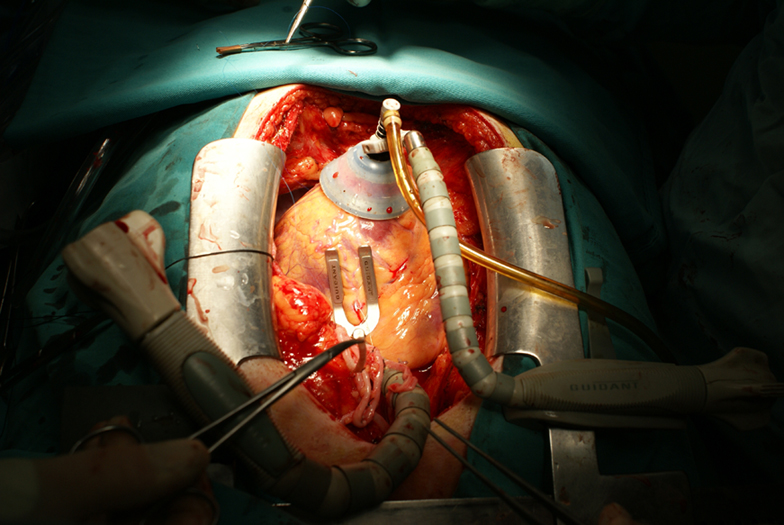
**A combined use of an apical vacuum positioning device and a heart stabilization device**.

**Figure 4:**
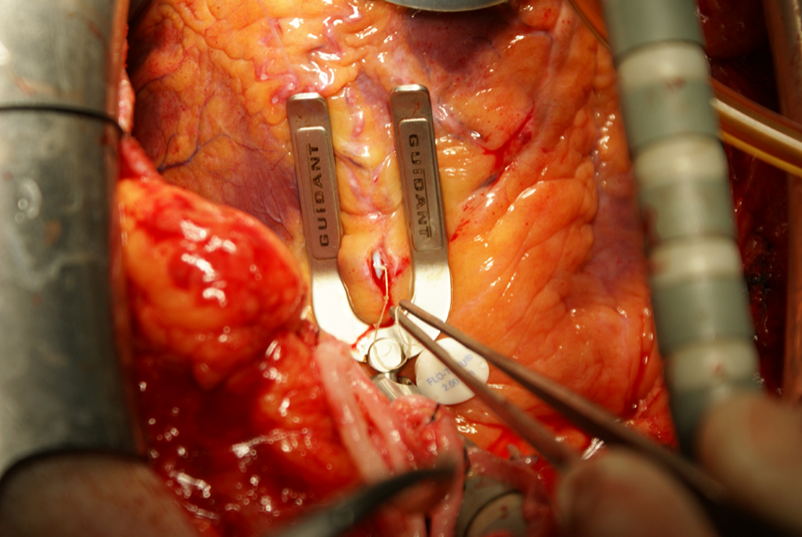
**A combined use of a heart stabilization device and an intra-coronary shunt device**.

If on-pump surgery was chosen, we administered 300 IU/kg heparin before routine aortic and venous cannulation to achieve the activated clotting time (ACT) between 400-600 seconds, and the operation was performed by using conventional on-pump technique. Myocardial protection was achieved by intermittent antegrade cold blood cardioplegia and topical ice slush. If the decision was off-pump technique, deep pericardial sutures and cardiac stabilization/positioning devices were employed to elevate and rotate the heart when required. During this investigation, we preferred to use Guidant cardiac stabilizator and apical vacuum device (Guidant Corporation, Santa Clara, CA, USA) (Figure 3). Coronary anastomoses were performed using intra-coronary shunt devices (Flo-Thru Intraluminal Shunt, Synovis Life Technologies, Inc, MN, USA) (Figure 4). If coronary artery has intensive atherosclerotic plaques, intra-coronary shunt devices were not used to avoid an iatrogenic intra-coronary plaque dissection, and a soft silastatic tourniquet and warm isotonic saline solution were used to obtain a bloodless anastomotic field. Intravenous beta-blockers were administered to achieve a target heart rate of 60-80 bpm and systolic blood pressure of 80-100 mmHg when required. In some cases, especially while during circumflex artery anastomosis, borderline hemodynamic status may not allow beta-blocker use.

Preoperative and postoperative cardiac enzyme levels including Troponin I, time interval between the onset of acute coronary syndrome and the operation, duration of the surgery, graft types and numbers, stay of intensive care unit and hospital stay were recorded. Incidence of atrial fibrillation, use of inotropes, use of blood products, and time to extubation were measured.

### Statistical analysis

Results are presented as mean ± SD for continuous variables and percent total for categorical variables. Comparisons of baseline characteristics were made with an independent-samples *t *test for continuous variables and χ^2 ^or Fischer's exact test was used for categorical variables. The odds ratio (OR) and 95% confidence intervals (CI) were given. All statistics were calculated with the use of the Statistical Package for Social Sciences (SPSS) for Windows, version 16.0.

## Results

Results are displayed in Table 1 and 2. There were no statistically significant differences between two groups considering age, gender, old myocardial infarction and other demographical variables. Although time interval from the onset of chest pain till the surgery was similar, number of patient operated within less than 6 hour after onset of chest pain was significantly higher in off pump group (76.1% versus 60.7% respectively, p = 0.036). That means the early admission to hospital, more chance to be operated via off-pump technique. EuroSCORE was lower in patients operated by off-pump technique than in patients who underwent on-pump surgery (p < 0.001). Need for intra-aortic balloon pump was lower in OPCAB group than on-pump group (3.52% and 12.5% respectively, p = 0.053). In other words, the patients who needed intra-aortic balloon pumping are candidates for on-pump technique.

**Table 2:** Preoperative, operative and postoperative variables of the patients.

	Off-pump (n = 142)	On-pump (n = 56)	Odds ratio	P value
**Onset of chest pain (hour)**	5.53 (± 1.47)	5.94 (± 1.99)	-	0.113
**Chest pain ≤ 6 h**	76.1%	60.7%	1.253	0.036
**Chest pain > 6 h**	23.9%	37.5%	0.638	0.080
**Angiography/PCI to operation time (minute)**	75.23 (± 23.53)	73.62 (± 21.34)	-	0.582
**Euroscore**	5.47 (± 1.64)	7.08 (± 2.15)	-	0.000
**Ejection fraction (%)**	43.08 (± 7.61)	42.75 (± 8.52)	-	0.788
**IABP**				
• **Preoperative insertion**	2 patients (1.4%)	2 patients (3.6%)	0.377	0.053
• **Postoperative insertion**	3 patients (2.1%)	5 patients (8.9%)	0.986	
**Postoperative inotrope (%)**	9.2	16.1	0.526	0.208
**Postoperative atrial fibrillation (%)**	10.6	19.6	0.483	0.104
**Peak Troponin-I level prior CABG (ng/mL)**	2.05 (± 1.88)	2.56 (± 2.02)	-	0.096
**Number of grafts**	2.57	2.73	-	0.166
• **One graft**	8 (5.6%)	2 (3.6%)		0.058
• **Two grafts**	54 (38%)	17 (30.4%)		0.000
• **Three grafts**	70 (49%)	31 (55.4%)		0.000
• **Four grafts**	10 (7%)	6 (10.7%)		0.317
**Operation time (min)**	82.78 (± 18.47)	164.22 (± (27.12)	-	0.000
**ICU stay (day)**	1.16 (± 0.36)	1.35 (± 0.58)	-	0.006
**Hospital stay (day)**	6.13 (± 1.37)	7.28 (± 1.61)	-	0.000
**Conversion to on-pump**	0	-	-	-
**Surgical revision**	2 patients (1.4%)	2 patients (3.6%)	0.386	0.317
**Mortality**	5 patients (3.5%)	3 patients (5.4%)	0.645	0.690

IABP: Intra-aortic balloon pump; PCI: Percutaneous coronary intervention; CABG: coronary artery bypass grafting; ICU: Intensive care unit.

Peak Troponin I level and preoperative/postoperative inotropic agent requirement were similar in both groups. Total graft number is same in both group (p = 0.166). However, the number of patients who had only one and two grafts was higher in off-pump group (p = 0.058 and p < 0.001 respectively), and the number of patients who had three grafts was higher in on-pump group (p < 0.001). There is not any significant difference between the number of the patients receiving four grafts (p = 0.317). Complete revascularization was achieved in all patients.

Operation time was shorter in off-pump group than on-pump group (82.78 min versus 164.22 min respectively, p < 000.1). Intensive care unit stay and hospital stay is were shorter in off-pump group than on pump group. On the other hand, there was not any statistically significant difference in in-hospital mortality rates (3.5% in off-pump group versus 5.4% in on-pump group). None of the patients were converted to on-pump surgery and none of the patients were converted to off-pump technique (e.g., because of calcified aorta, etc.).

## Discussion

Because of known deleterious effects of cardiopulmonary bypass, off-pump surgery has been preferred by many skilled surgeons. At the beginning, performing OPCAB surgery was more difficult than present. Parallel to development in surgical instruments (proximal anastomotic devices, suction stabilizers, apical cup-suction devices, intra-coronary shunts), off-pump skills and techniques improved by the years. By a controversy, besides technical difficulties such as heart positioning, maintaining sufficient hemodynamic status, bloody surgical field etc, emergent conversion to extracorporeal circulation is associated to high mortality and morbidity [7,10,14]. In some cardiac centers, OPCAB lost its interest short after such unpleasant experiences. Therefore, we need a safe and useful algorithm to complete OPCAB avoiding conversion. Maybe the most important result of this study is that none of the patients in OPCAB group required conversion to on-pump technique.

Recently, many investigations have been published to compare off-pump and on-pump methods [4,6-9,11,12,15,16]. In fact, only a few centers reported their results that OPCAB trials had been succeeded without conversion to on pump. Besides, it has been well known that the aborted off-pump patients have significantly higher risks of operative mortality and morbidity than those which are completed off-pump [10]. The most frequent problem while performing OPCAB surgery is to provide an adequate hemodynamic condition. There are different techniques and equipments to position the heart and stabilize the operative field. Deep pericardial sutures, heart retraction tapes, wet sponges suspenders underneath the heart were used previously. Recently, newly designed positioning and stabilizing devices are in use. These devices help to keep stable hemodynamic condition during performing coronary anastomoses as well as stabilizing the heart. Unfortunately, they may not provide adequate hemodynamics in some cases even though patient has good left ventricular functions.

Besides timing of surgery in case of ongoing acute coronary syndrome, preferential surgical technique is another important point to get a satisfactory result. Locker and colleagues advocate avoiding cardiopulmonary bypass because it is associated with lower operative mortality for emergency patients operated within the first 48 hours of symptom after onset [13]. First six hours after acute myocardial infarction is known as "golden hours" and previous studies have reported mortality rates between 3.1% and 11.8% during this period [17]. It also has been reported that operative mortality of patients with acute myocardial infarction undergone to emergent off-pump surgery was 5%, compared to 24% in patients operated with on-pump [18]. In our study we found a lower mortality rate in off-pump group than on-pump patients (3.5% and 5.4% respectively) but it does not have statistical significance (p = 0.690). We think the most important factor associated with low mortality rates in both groups was the early operation time. We operated 72.2% of patients within first 6 hours after onset of chest pain. A previously published article by Nunley has reported the mortality rate of AMI patients who underwent on-pump surgery within the first 48 hours of AMI was 7.7%, compared with 0% after 48 hours [19]. Lee et al have reported similar mortality rates [17]. Our results showed that the patients who admitted to the operating room within first 6 hours after onset of chest pain were more suitable to meet our off-pump surgery criteria. 76.1% of the patients who were operated within first 6 hours after onset of chest pain underwent off-pump surgery, but comparably less patients (60.7%) had the chance to get off pump criteria if they were operated after 6^th ^hour (p = 0.036, odds ratio = 1.253). Therefore, it can be interpreted that patients receiving hospital within "golden hours" have a greater chance to be a candidate for off-pump surgery. We think this condition is associated with the short ischemic time.

It is interesting that although some patients with low Ejection Fraction (EF) well tolerated heart positioning (e.g. while doing circumflex artery bypass) during off pump surgery others with normal EF could not maintain hemodynamic status while handling the heart and needed medical support. It was previously reported that off-pump coronary surgery could be preferred with an acceptable mortality for the risky patients who have an EF lower than 30% [20]. In our study, although EF levels were similar, need for IABP after surgery was significantly higher in on-pump group (p = 0.053). A problem that emerges during off-pump surgery is management of the blood pressure; what can we do? Some cardiac centers do not use any cardiac positioning and/or stabilization device, and some of them abandoned performing off-pump surgery because of a high rate of conversion to on-pump due to inconvenient hemodynamics. Apical suction devices serve a good exposure for circumflex artery and its branches without collapsing left ventricular cavity, and it can be combined with a stabilizing device to achieve more comfortable condition by stabilizing the field of anastomosis on the heart. We routinely used a cardiac stabilizing device in combination with an apical suction positioning device and an intra-coronary shunt (Figures 1 and 2).

Our results show that, on-pump group has significantly higher EuroSCORE levels than OPCAB group at admission. However, EuroSCORE did not affect to patient selection criteria. Although a careful scoring prior the surgery, the surgeon did not make a decision according to patients' EuroSCORE on deciding the surgical technique. This can be commented as the higher EUROSCORE means the more requirement for extracorporeal circulatory assist. More definite data should be collected via a prospective randomized research to prove, if present, any relationship between EUROSCORE and on pump surgery.

Beta-receptor blocking agents are preferential drugs to manage heart rate and blood pressure during OPCAB. They may also prevent myocardial ischemia in a more or less hyperdynamic heart. Nevertheless, sometimes slowing down the heart rate and lowering the blood pressure can cause severe hypotension especially while exposing circumflex system. This hypotensive episode can cause to exacerbate ischemia reducing blood flow beyond stenosed or obstructed coronary arteries, and emergent conversion to on-pump may become inevitable. To avoid this catastrophic event, we hesitate to administer beta-blocker unless systolic blood pressure exceeds 150 mmHg. So handling the heart to give intended position becomes safer even if circumflex artery anastomosis.

Szygula-Jurkiewicz and colleagues have found a better 1-year physical functioning for the CABG patients compared with the percutaneous coronary intervention (PCI) patients [21]. They reported that their patients in the PCI group had more frequent episodes of unstable angina and a higher rate of repeat revascularization during 1-year follow-up. In our study, during the coronary angiography session we made a rapid evaluation together with our cardiologists and decided to perform PCI or coronary artery bypass surgery. 391 patients were directed to PCI according to their coronary angiography and clinical status, but 12 of them had acute stent thrombosis and/or failed PCI, and they underwent emergent coronary surgery.

On the other hand, our results supported that off-pump surgery has some well-known advantages over on-pump surgery; significantly lower rate of IABP support than on-pump group (3.5% versus 12.5%, p = 0.053), shorter operation time (82.78 min versus 164.22 min, p < 0.001), ICU stay (p < 0.05) and hospital stay (p < 0.001). Besides, although we did not measure neurocognitive functions, we observed that off-pump patients were more interested in their environment and they were more cooperated and oriented. Off-pump patients were able to mobilize themselves easier than on-pump patients.

Beneficial effects of off-pump surgery were declared many times in the past. However, it is also known that results of acute conversion to on-pump surgery are more complicated. Although most of cardiac surgeons believe the benefits of off pump surgery, some does not prefer to do this operation. With this investigation, we aimed to find a successful and safe method for off-pump coronary surgery that can be easily applied by cardiac surgeons, and to share our results. As a summary, following the pathway step-by-step described in Figure 2, we propose a safe decision-making method for selecting the suitable candidate for off-pump surgery. So, off-pump coronary artery surgery technique may become more prevalent in general use. Retrospective study design and lack of random assignment are the major limitations of this study, and we still need a larger and multicenter investigation to improve off-pump surgical strategies.

## Competing interests

The authors declare that they have no competing interests.

## Authors' contributions

**KK: **participated in the design, coordinated the study, performed the statistical analysis. **RC: **participated in the design of the study and the statistical analysis. **AT: **helped to draft the manuscript. **MFTS: **participated in the design of the study. **AA**: participated in the design of the study and the statistical analysis. **GG: **helped to draft the manuscript. **SM: **performed coronary angiographies, participated in the design of the study. **RT: **participated in the design of the study. All authors read and approved the final manuscript.
